# Influence of Gender and Skin Color on the Perception of Tooth Shades in a Moroccan Subpopulation

**DOI:** 10.7759/cureus.107666

**Published:** 2026-04-24

**Authors:** Hanane El Bekkali, Imane El Bassity, Nezha Ben El Hammi, Farouk Mohammed, El Mehdi Jouhadi

**Affiliations:** 1 Department of Fixed Prosthodontics, Faculty of Dental Medicine, Hassan II University of Casablanca, Casablanca, MAR; 2 Department of Dentistry, Sidi Mohamed Ben Abdellah University, Fes, MAR

**Keywords:** dental esthetics, fitzpatrick scale, skin color, smile esthetics, tooth shade

## Abstract

Introduction and aim

Dental esthetics is a key component of modern dentistry, with tooth shade playing a major role in smile harmony. This study aimed to evaluate the influence of skin color and gender on the perception of tooth shades within a Moroccan population.

Methods

In this cross-sectional study, 300 non-dental participants were recruited using a convenience sampling method. Participants evaluated 48 digitally modified smile photographs of one male and one female model. Six tooth shades (BL1-A1) were combined with four Fitzpatrick skin types (II-V) using spectrophotometric calibration based on the CIE L*a*b* color space. Participants selected the most esthetically pleasing shade for each skin type. Associations between shade preference and demographic variables were analyzed using Pearson's chi-square tests, with Monte Carlo permutation (B = 100,000 simulations) applied when more than 20% of expected cell counts were below five. Effect sizes were assessed using Cramer's V coefficient.

Results

Lighter shades (BL1-BL3) were predominantly selected across all skin types and demographic subgroups. No statistically significant association was observed for gender, age, or professional status (p > 0.05). Education level showed statistically significant but weak associations (Cramer's V = 0.19-0.21). Overall, lighter shades accounted for 69-92% of selections.

Conclusion

In this Moroccan sample, lighter tooth shades were predominantly preferred across all skin types and observer subgroups. These findings suggest a general tendency toward higher-value (brighter) tooth shades in esthetic perception, although no causal inference can be made. Individualized shade selection remains essential in clinical practice.

## Introduction

Dental esthetics is a multifactorial concept influenced by several parameters, among which the esthetic triad of shape, color, and alignment plays a fundamental role in achieving facial harmony [1]. Among these factors, tooth color is particularly complex, as it is determined by both intrinsic and extrinsic elements, including tooth translucency, opacity, lighting conditions, and light scattering, all of which influence visual perception [2].

The selection of an appropriate tooth shade is therefore a critical component of esthetic dentistry, directly impacting the integration of restorations and prosthetic rehabilitations [3]. This choice is further influenced by individual and cultural factors such as skin tone, gender, age, and societal preferences. Several studies have reported an association between tooth shade and skin color, with lighter skin tones frequently linked to lighter tooth shades to achieve optimal esthetic harmony [4]. However, conflicting results have also been reported. Some authors observed that individuals with darker skin tones may prefer lighter shades [5,6], whereas others found a significant association between lighter skin phototypes and lighter tooth shades, or no consistent relationship at all [7,8]. These discrepancies highlight the need for population-specific data, particularly in regions with wide variation in skin phototype, such as North Africa and the Middle East.

In Morocco, where skin phototypes range widely from Fitzpatrick types II to V, tooth shade selection in clinical practice is commonly guided by standardized international shade systems, such as the VITA Classical and VITA 3D-MASTER systems. However, these systems are based on universal color standards and do not specifically account for population-based variations in esthetic perception.

While smile esthetics have been investigated in various populations, including European and Asian populations, as well as a limited number of Middle Eastern and African cohorts, limited data are available regarding the influence of gender and skin tone on the perception of the most esthetically pleasing tooth shades in the Moroccan population.

Therefore, the aim of this cross-sectional study was to evaluate the influence of model skin color and observer characteristics, including gender, age, education level, and professional status, on tooth shade preference using digitally standardized smile photographs modified according to the Ivoclar Vivadent A-D shade guide and Fitzpatrick skin types. By analyzing participant preferences and their association with demographic variables, this study seeks to provide clinically relevant insights to guide tooth shade selection in esthetic dental practice tailored to the Moroccan population.

## Materials and methods

Study design

This cross-sectional, single-center observational study was conducted at the Ibn Rochd Center for Dental Consultation and Treatment in Casablanca, Morocco. The study used digitally standardized smile photographs modified to represent different tooth shades and skin tones.

Digital image preparation

**Figure 1:**
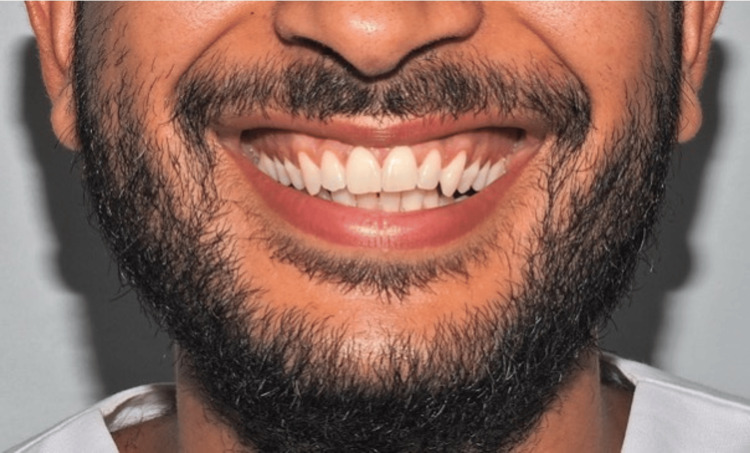
Male smile photograph.

**Figure 2:**
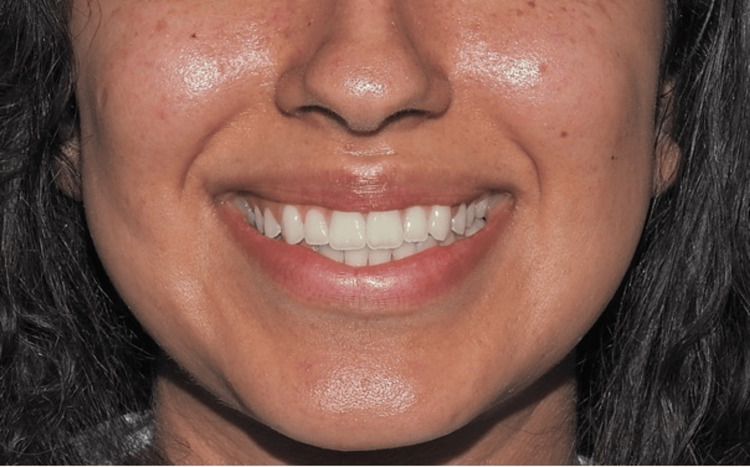
Female smile photograph.

The following two adult models were selected: one male (24 years old) and one female (23 years old), both presenting esthetically harmonious smiles. Informed consent was obtained from both models prior to their participation. Standardized frontal photographs, including the perioral region, were taken using a digital camera (Nikon D90 with flash {Tokyo, Japan: Nikon Corp.}), positioned at the level of the occlusal plane to ensure reproducibility (Figures 1, 2).

Tooth shades were digitally modified according to the Ivoclar Vivadent A-D shade guide using the following six shades: BL1, BL2, BL3, BL4, B1, and A1. Color calibration was performed using an electronic spectrophotometer (SpectroShade Micro; Verona, Italy: MHT S.p.A.). Each shade tab was recorded in International Commission on Illumination L*a*b* (CIELAB) coordinates (L*: lightness; a*: green-red axis; b*: blue-yellow axis). These coordinates were applied to the teeth using GIMP software (GIMP Development Team) to generate standardized digital modifications.

Skin tone modification

**Figure 3:**
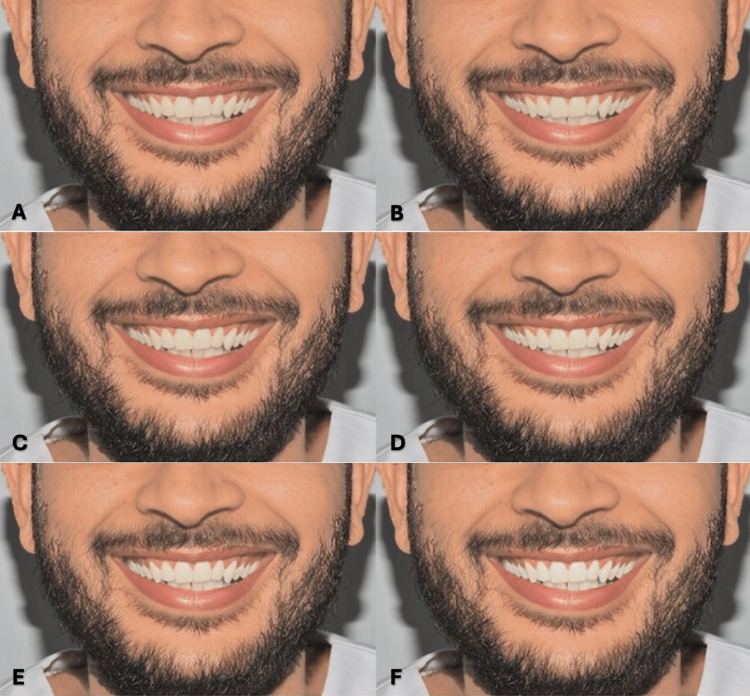
Clinical photograph of a male participant presenting type II skin color. The image shows (A) tooth shade A1, (B) tooth shade B1, (C) tooth shade BL4, (D) tooth shade BL3, (E) tooth shade BL2, and (F) tooth shade BL1.

**Figure 4:**
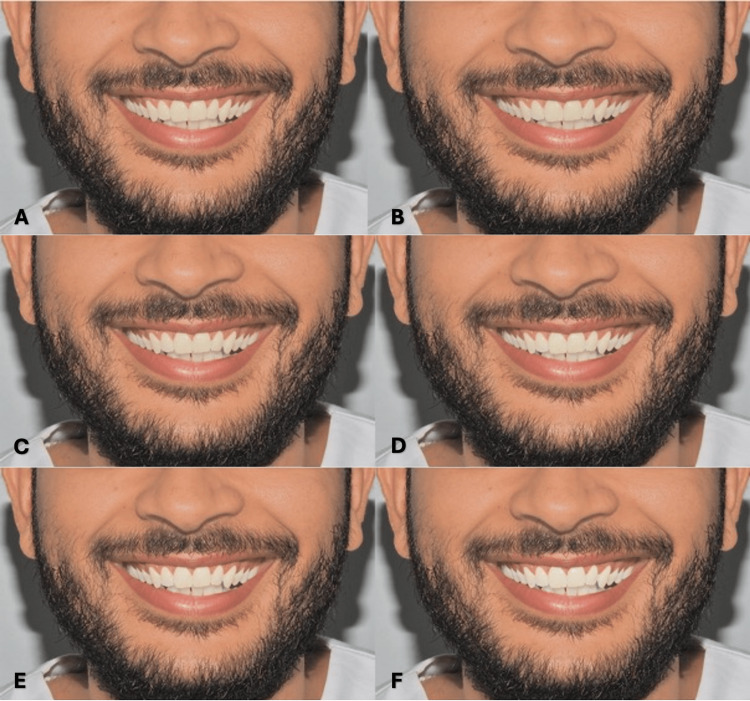
Clinical photograph of a male participant presenting type III skin color. The image shows (A) tooth shade A1, (B) tooth shade B1, (C) tooth shade BL4, (D) tooth shade BL3, (E) tooth shade BL2, and (F) tooth shade BL1.

**Figure 5:**
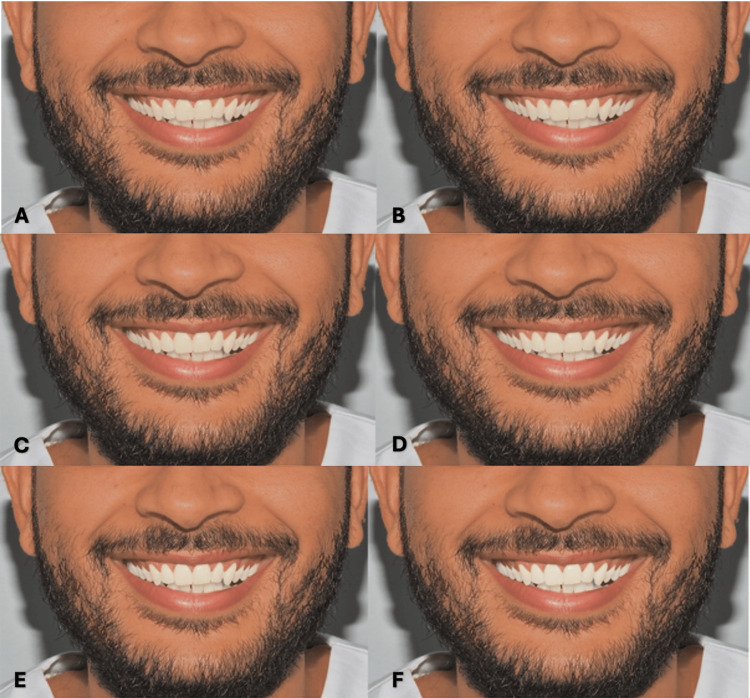
Clinical photograph of a male participant presenting type IV skin color. The image shows (A) tooth shade A1, (B) tooth shade B1, (C) tooth shade BL4, (D) tooth shade BL3, (E) tooth shade BL2, and (F) tooth shade BL1.

**Figure 6:**
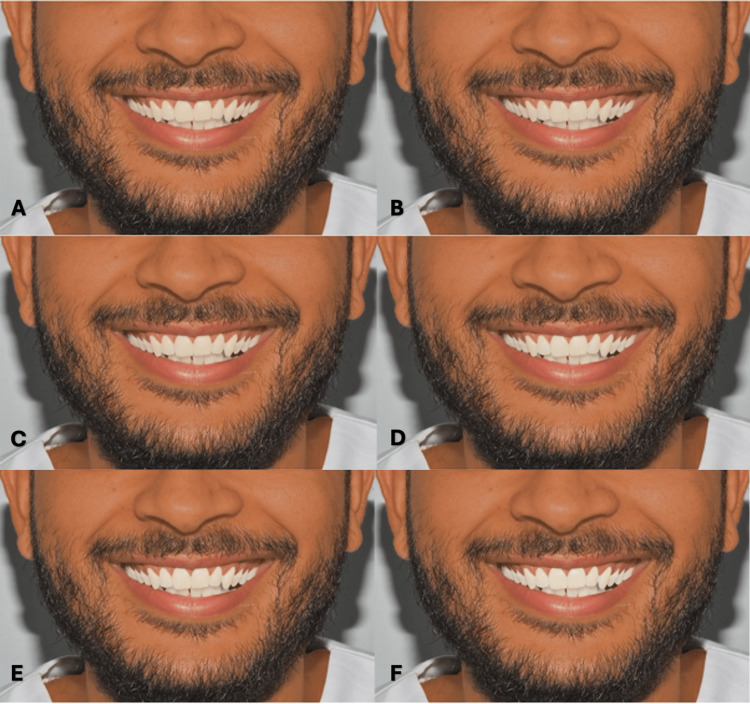
Clinical photograph of a male participant presenting type V skin color. The image shows (A) tooth shade A1, (B) tooth shade B1, (C) tooth shade BL4, (D) tooth shade BL3, (E) tooth shade BL2, and (F) tooth shade BL1.

**Figure 7:**
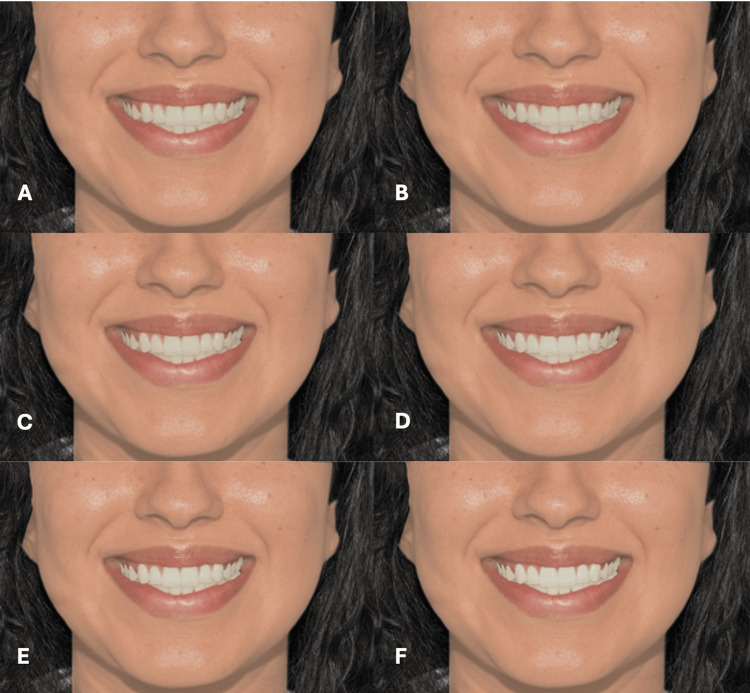
Clinical photograph of a female participant presenting type II skin color. The image shows (A) tooth shade A1, (B) tooth shade B1, (C) tooth shade BL4, (D) tooth shade BL3, (E) tooth shade BL2, and (F) tooth shade BL1.

**Figure 8:**
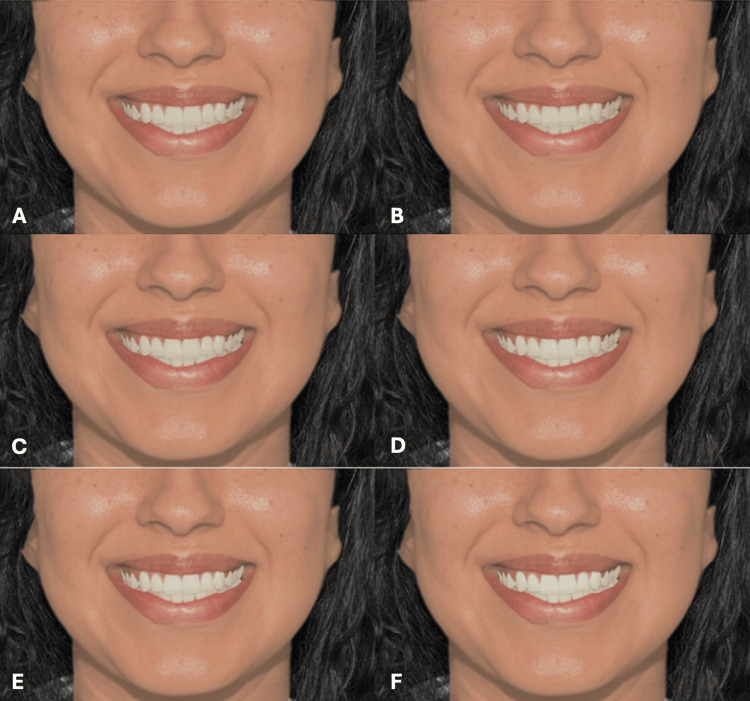
Clinical photograph of a female participant presenting type III skin color. The image shows (A) tooth shade A1, (B) tooth shade B1, (C) tooth shade BL4, (D) tooth shade BL3, (E) tooth shade BL2, and (F) tooth shade BL1.

**Figure 9:**
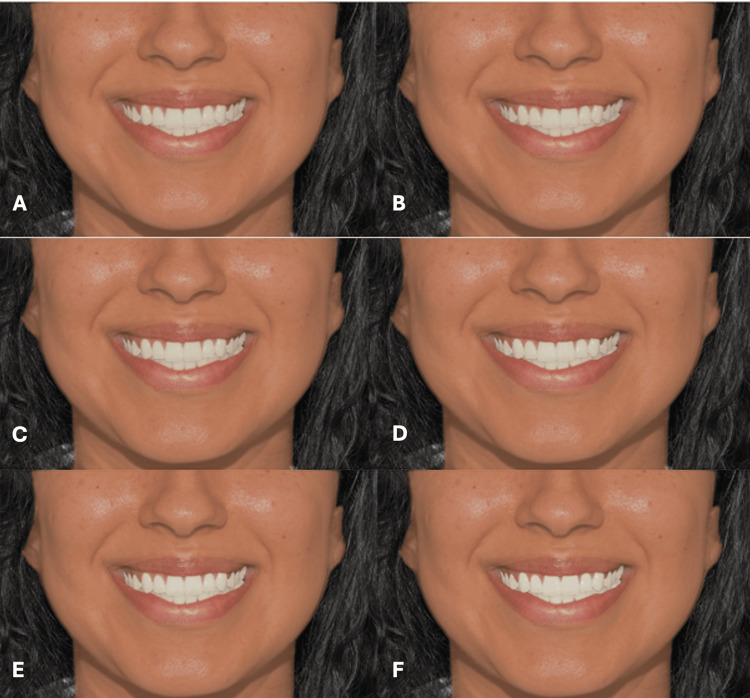
Clinical photograph of a female participant presenting type IV skin color. The image shows (A) tooth shade A1, (B) tooth shade B1, (C) tooth shade BL4, (D) tooth shade BL3, (E) tooth shade BL2, and (F) tooth shade BL1.

**Figure 10:**
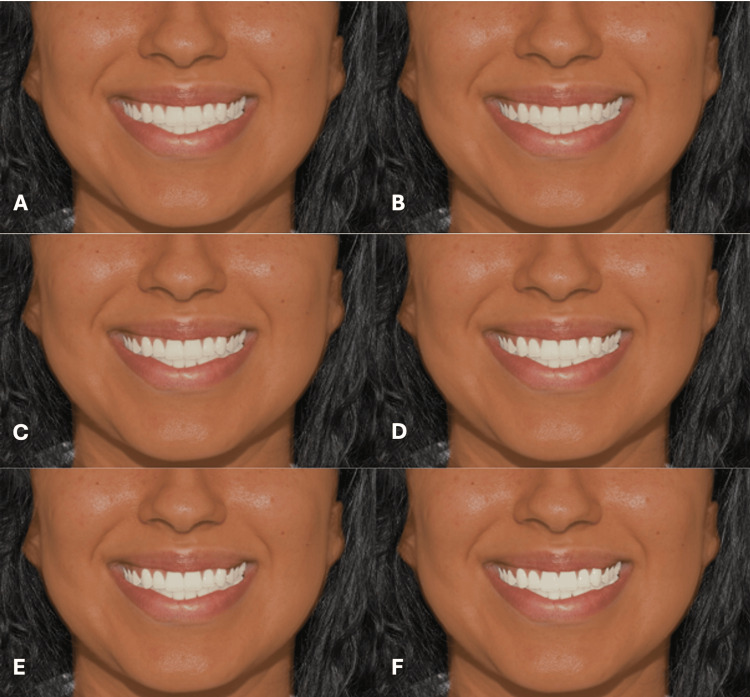
Clinical photograph of a female participant presenting type V skin color. The image shows (A) tooth shade A1, (B) tooth shade B1, (C) tooth shade BL4, (D) tooth shade BL3, (E) tooth shade BL2, and (F) tooth shade BL1.

For each model, skin color was digitally modified according to the following four Fitzpatrick skin types: type II (fair), type III (medium), type IV (olive), and type V (light brown) [9]. Each skin type was combined with the six tooth shades, resulting in 48 images (two models × four skin types × six shades). All images were standardized in size, brightness, and contrast (Figures 3A-3F, 4A-4F, 5A-5F, 6A-6F, 7A-7F, 8A-8F, 9A-9F, 10A-10F).

Pilot validation

A two-step pilot procedure was conducted to verify the visual concordance between the digitally modified tooth shades and the corresponding physical shade tabs from the A-D shade guide. In the first pilot phase, 10 dentists independently evaluated the correspondence between the digitally modified photographs and the original shade tabs. This initial evaluation revealed minor discrepancies between the intended CIELAB values and the visual perception of the modified images.

Following correction of the color calibration, a second pilot phase was conducted, including the same 10 dentists and 10 additional dentists. Participants again matched the digitally modified tooth shades with the corresponding physical shade tabs.

This pilot procedure was used to refine the digital calibration and improve the visual consistency between the modified images and the reference shade tabs prior to the main data collection. No formal agreement statistics were calculated, as the pilot phase was conducted primarily for calibration and methodological refinement rather than for assessment of inter-observer reliability.

Participants

A total of 300 participants were recruited based on feasibility considerations and in accordance with sample sizes reported in previous studies on dental esthetic perception. Inclusion criteria were age ≥18 years, absence of professional dental training, and normal color vision. Exclusion criteria included dentists, individuals with color vision deficiency, and participants under 18 years of age. Color vision was screened using the Ishihara test [10]. No participants were excluded due to color vision deficiency. Informed consent was obtained from all participants.

Questionnaire and evaluation procedure

Participants completed a demographic questionnaire including gender, age, education level, occupation, and income (appendix 1). Participants also rated their satisfaction with their smile and the importance of smile esthetics using a visual analog scale (VAS 0-10). Each participant was presented with four sets of photographs corresponding to the four Fitzpatrick skin types. Each set contained six images representing the six tooth shades. Participants were asked to select the most esthetically pleasing tooth shade for each skin tone. Images were displayed on an Apple iPad Pro (Cupertino, CA: Apple Inc.) with brightness set to 100% to ensure standardized viewing conditions. All assessments were conducted in a controlled indoor environment under consistent ambient lighting and standardized viewing distance. A test-retest procedure was not performed, as the evaluation was designed as a single-session perceptual task to minimize recall bias. All responses were recorded immediately after selection using a standardized data collection form and subsequently coded and entered into a secure database for statistical analysis.

Statistical analysis

Data were analyzed using Jamovi version 2.6 software (Sydney, Australia: Jamovi project). Categorical variables were summarized using frequencies and percentages. Associations between tooth shade preference and demographic variables were assessed separately for each of the four Fitzpatrick skin types (II-V) and model gender using Pearson's chi-square test.

When more than 20% of expected cell counts were below five, p-values were calculated using chi-square tests with Monte Carlo permutation (B = 100,000 simulations). Effect sizes were evaluated using Cramer's V coefficient, interpreted as follows: values of 0.10-0.20 indicate a weak association, 0.20-0.40 a moderate association, and > 0.40 a strong association.

Additionally, a secondary analysis was performed by grouping tooth shades into lighter shades (BL1-BL4) and conventional shades (A1-B1). Associations between grouped shade preference and demographic variables were assessed using chi-square tests. A significance level of p < 0.05 was adopted.

## Results

Participant characteristics

A total of 300 participants were included in this study. The sample consisted of 91 males (30.3%) and 209 females (69.7%). Age distribution was as follows: 18-34 years (36%), 35-49 years (39%), and 50-70 years (25%). Education levels were distributed as illiterate (17%), primary (11.7%), middle school (17%), high school (18.7%), and university (35.7%). A large proportion of participants declined to disclose income; therefore, this variable was excluded from further analysis. Variables related to smile satisfaction and perceived importance of smile esthetics were collected descriptively but were not included in the inferential statistical analysis, as they were not part of the primary study objectives (Table 1).

**Table 1:** Distribution of sociodemographic characteristics of participants (n = 300).

Characteristics	n (%)
Gender	
Male	91 (30.3%)
Female	209 (69.7%)
Age groups	
18-34 years	108 (36%)
35-49 years	117 (39%)
50-70 years	75 (25%)
Education level	
Illiterate	51 (17%)
Primary	35 (11.7%)
Middle school	51 (17%)
High school	56 (18.7%)
University	107 (35.7%)
Occupation	
Unemployed	140 (46.7%)
Governmental employee	95 (31.7%)
Private employee	65 (21.7%)

Distribution of selected tooth shades

The distribution of preferred tooth shades by Fitzpatrick skin type and by the model's and observer's gender is presented in Table 2. Across Fitzpatrick skin types II through V, lighter shades (BL1, BL2, and BL3) were most frequently selected. For the female model, BL1 and BL3 were the most commonly selected shades, whereas for the male model, BL1 and BL2 predominated. The chi-square goodness-of-fit test indicated that shade selections were not uniformly distributed within each skin type (all p < 0.001), suggesting a general preference for lighter tooth shades (appendix 2).

**Table 2:** Frequency of dental shade selection by Fitzpatrick skin type and model gender. Data are presented as n (%). The chi-square goodness-of-fit test (df = 5) evaluated whether shade selections were uniformly distributed across the six shade options within each group (n = 300 per group). All minimum expected cell counts were ≥ 50; the standard Pearson's chi-square test was applied throughout. BL1, BL2, BL3, BL4, B1, and A1 refer to tooth shade tabs from the Ivoclar Vivadent A-D shade guide. All p < 0.001.

Model/skin type	BL1, n (%)	BL2, n (%)	BL3, n (%)	BL4, n (%)	B1, n (%)	A1, n (%)	χ²	p-Value
Female - type II	100 (33.3)	56 (18.7)	80 (26.7)	38 (12.7)	16 (5.3)	10 (3.3)	126.72	<0.001
Female - type III	79 (26.3)	70 (23.3)	71 (23.7)	40 (13.3)	28 (9.3)	12 (4.0)	74.20	<0.001
Female - type IV	75 (25.0)	42 (14.0)	55 (18.3)	42 (14.0)	60 (20.0)	26 (8.7)	29.08	<0.001
Female - type V	81 (27.0)	37 (12.3)	51 (17.0)	39 (13.0)	58 (19.3)	34 (11.3)	31.44	<0.001
Male - type II	121 (40.3)	72 (24.0)	49 (16.3)	35 (11.7)	13 (4.3)	10 (3.3)	174.40	<0.001
Male - type III	92 (30.7)	73 (24.3)	67 (22.3)	40 (13.3)	21 (7.0)	7 (2.3)	107.44	<0.001
Male - type IV	87 (29.0)	57 (19.0)	68 (22.7)	30 (10.0)	35 (11.7)	23 (7.7)	61.92	<0.001
Male - type V	74 (24.7)	52 (17.3)	65 (21.7)	38 (12.7)	42 (14.0)	29 (9.6)	29.08	<0.001

Influence of observer gender

No statistically significant association was observed between observer gender and preferred tooth shade for either the female or male model across Fitzpatrick skin types II through V (all p > 0.05) (Table 3). Both male and female observers demonstrated similar preference patterns, with lighter shades selected more frequently in both groups.

**Table 3:** Distribution and comparison of preference of the six dental shades in each of the four Fitzpatrick skin type groups among male and female participants. *P-value calculated using chi-square with Monte Carlo permutation test (B = 100,000 simulations) due to expected cell counts < 5 in one or more cells (minimum expected count: female model types II-III, min E = 3.0-3.6; male model types II-III, min E = 2.1-3.0). Data are presented as n (%). Statistical comparisons between female and male observers within each skin type were performed using Pearson's chi-square test (df = 5). No statistically significant difference was observed between male and female observers for any skin type or model (all p > 0.05). BL1, BL2, BL3, BL4, B1, and A1 refer to tooth shade tabs from the Ivoclar Vivadent A-D shade guide. F: female; M: male; min E: minimum expected cell count in the contingency tables

Model/skin type	Observer gender	BL1, n (%)	BL2, n (%)	BL3, n (%)	BL4, n (%)	B1, n (%)	A1, n (%)	χ²	p-Value
Female model									
Type II	F	66 (21.9)	39 (12.9)	64 (21.3)	26 (8.6)	8 (2.6)	6 (1.9)	8.08	0.779*
	M	34 (11.3)	17 (5.6)	16 (5.3)	12 (3.9)	8 (2.6)	4 (1.3)		
Type III	F	51 (16.9)	49 (16.3)	54 (17.9)	26 (8.6)	22 (7.3)	7 (2.3)	4.54	0.928*
	M	28 (9.3)	21 (6.9)	17 (5.6)	14 (4.6)	6 (1.9)	5 (1.6)		
Type IV	F	48 (15.9)	30 (9.9)	41 (13.6)	32 (10.6)	41 (13.6)	17 (5.6)	2.94	0.709
	M	27 (8.9)	12 (3.9)	14 (4.6)	10 (3.3)	19 (6.3)	9 (2.9)		
Type V	F	48 (15.9)	23 (7.6)	38 (12.6)	32 (10.6)	45 (14.9)	23 (7.6)	10.32	0.067
	M	33 (10.9)	14 (4.6)	13 (4.3)	7 (2.3)	13 (4.3)	11 (3.6)		
Male model									
Type II	F	76 (25.3)	57 (18.9)	35 (11.6)	28 (9.3)	6 (1.9)	7 (2.3)	11.01	0.627*
	M	45 (14.9)	15 (4.9)	14 (4.6)	7 (2.3)	7 (2.3)	3 (0.9)		
Type III	F	59 (19.6)	58 (19.3)	51 (16.9)	23 (7.6)	14 (4.6)	4 (1.3)	9.37	0.707*
	M	33 (10.9)	15 (4.9)	16 (5.3)	17 (5.6)	7 (2.3)	3 (0.9)		
Type IV	F	59 (19.6)	39 (12.9)	47 (15.6)	24 (7.9)	29 (9.6)	11 (3.6)	9.78	0.082
	M	28 (9.3)	18 (5.9)	21 (6.9)	6 (1.9)	6 (1.9)	12 (3.9)		
Type V	F	45 (14.9)	37 (12.3)	49 (16.3)	30 (9.9)	25 (8.3)	23 (7.6)	8.68	0.123
	M	29 (9.6)	15 (4.9)	16 (5.3)	8 (2.6)	17 (5.6)	6 (1.9)		

Influence of age

The chi-square test revealed no statistically significant association between age group and tooth shade preference in most comparisons (Table 4). A single statistically significant association was observed in the female model with Fitzpatrick skin type V (χ² = 20.73, p = 0.023). In this subgroup, participants aged 18-34 years more frequently selected shade B1, whereas participants aged 35-49 years and 50-70 years selected BL1 more frequently. No consistent age-related pattern was observed across other skin types or for the male model.

**Table 4:** Distribution of tooth shade selections by Fitzpatrick skin type, observer gender, and age group, with chi-square test results. *P-value calculated using chi-square with Monte Carlo permutation test (B = 100,000 simulations) due to expected cell counts < 5 in one or more cells. **P < 0.05 is statistically significant. Data are presented as n (%). Statistical comparisons across age groups (18-34, 35-49, 50-70 years) within each skin type were performed using Pearson's chi-square test (df = 10). Statistically significant p-values (p < 0.05) are shown in bold. BL1, BL2, BL3, BL4, B1, and A1 refer to tooth shade tabs from the Ivoclar Vivadent A-D shade guide.

Model/skin type	Age group, years	BL1, n (%)	BL2, n (%)	BL3, n (%)	BL4, n (%)	B1, n (%)	A1, n (%)	χ²	p-Value
Female model									
Type II	18-34	29 (9.6)	23 (7.6)	36 (11.6)	16 (5.3)	3 (0.9)	1 (0.3)	17.93	0.529*
	35-49	44 (14.6)	18 (5.9)	22 (7.3)	18 (5.9)	9 (2.9)	6 (1.9)		
	50-70	27 (8.9)	15 (4.9)	22 (7.3)	4 (1.3)	4 (1.3)	3 (0.9)		
Type III	18-34	20 (6.6)	27 (8.9)	30 (9.9)	21 (6.9)	9 (2.9)	1 (0.3)	21.68	0.374*
	35-49	39 (12.9)	19 (6.3)	29 (9.6)	12 (3.9)	12 (3.9)	6 (1.9)		
	50-70	20 (6.6)	24 (7.9)	12 (3.9)	7 (2.3)	7 (2.3)	5 (1.6)		
Type IV	18-34	19 (6.3)	20 (6.6)	19 (6.3)	21 (6.9)	21 (6.9)	8 (2.6)	12.75	0.238
	35-49	31 (10.3)	14 (4.6)	21 (6.9)	16 (5.3)	24 (7.9)	11 (3.6)		
	50-70	25 (8.3)	8 (2.6)	15 (4.9)	5 (1.6)	15 (4.9)	7 (2.3)		
Type V	18-34	19 (6.3)	13 (4.3)	20 (6.6)	21 (6.9)	27 (8.9)	8 (2.6)	20.73	0.023**
	35-49	32 (10.6)	15 (4.9)	19 (6.3)	13 (4.3)	20 (6.6)	18 (5.9)		
	50-70	30 (9.9)	9 (2.9)	12 (3.9)	5 (1.6)	11 (3.6)	8 (2.6)		
Male model									
Type II	18-34	38 (12.6)	34 (11.3)	17 (5.6)	16 (5.3)	3 (0.9)	0 (0)	15.45	0.649*
	35-49	48 (15.9)	23 (7.6)	22 (7.3)	13 (4.3)	6 (1.9)	5 (1.6)		
	50-70	35 (11.6)	15 (4.9)	10 (3.3)	6 (1.9)	4 (1.3)	5 (1.6)		
Type III	18-34	26 (8.6)	33 (10.9)	27 (8.9)	16 (5.3)	5 (1.6)	1 (0.3)	13.63	0.740*
	35-49	43 (14.3)	27 (8.9)	23 (7.6)	14 (4.6)	8 (2.6)	2 (0.6)		
	50-70	23 (7.6)	13 (4.3)	17 (5.6)	10 (3.3)	8 (2.6)	4 (1.3)		
Type IV	18-34	21 (6.9)	25 (8.3)	26 (8.6)	16 (5.3)	13 (4.3)	7 (2.3)	14.09	0.169
	35-49	43 (14.3)	22 (7.3)	24 (7.9)	8 (2.6)	12 (3.9)	8 (2.6)		
	50-70	23 (7.6)	10 (3.3)	18 (5.9)	6 (1.9)	10 (3.3)	8 (2.6)		
Type V	18-34	22 (7.3)	22 (7.3)	21 (6.9)	17 (5.6)	16 (5.3)	10 (3.3)	5.09	0.885
	35-49	33 (10.9)	17 (5.6)	27 (8.9)	14 (4.6)	16 (5.3)	10 (3.3)		
	50-70	19 (6.3)	13 (4.3)	17 (5.6)	7 (2.3)	10 (3.3)	9 (2.9)		

Influence of education level

A statistically significant association between education level and tooth shade preference was observed for the female model across all four Fitzpatrick skin types (type II: χ² = 51.77, p = 0.006; type III: χ² = 42.44, p = 0.038; type IV: χ² = 44.06, p = 0.028; type V: χ² = 52.19, p = 0.005) (Table 5). For the male model, significant associations were observed for types II (χ² = 43.77, p = 0.031) and III (χ² = 52.60, p = 0.005), whereas no statistically significant association was found for types IV and V.

**Table 5:** Distribution of tooth shade selections by Fitzpatrick skin type, observer gender, and education level, with chi-square test results. *P-value calculated using chi-square with Monte Carlo permutation test (B = 100,000 simulations) due to expected cell counts < 5 in one or more cells (minimum expected count: 0.8). This correction was applied to all subgroups in this table. **P < 0.05 is statistically significant. BL1, BL2, BL3, BL4, B1, and A1 refer to tooth shade tabs from the Ivoclar Vivadent A-D shade guide. Data are presented as n (%). All p-values were calculated using the chi-square test with Monte Carlo permutation (B = 100,000 simulations, df = 20), as multiple expected cell counts were < 5 across all education-level subgroups (minimum expected count: 0.8).

Model/skin type	Education level	BL1, n (%)	BL2, n (%)	BL3, n (%)	BL4, n (%)	B1, n (%)	A1, n (%)	χ²	p-Value
Female model									
Type II	Illiterate	26 (8.7)	8 (2.7)	9 (3.0)	3 (1.0)	2 (0.7)	3 (1.0)	51.77	0.006**
	Primary	15 (5.0)	9 (3.0)	5 (1.7)	3 (1.0)	1 (0.3)	2 (0.7)		
	Middle school	24 (8.0)	6 (2.0)	7 (2.3)	8 (2.7)	4 (1.3)	2 (0.7)		
	High school	15 (5.0)	9 (3.0)	19 (6.3)	4 (1.3)	7 (2.3)	2 (0.7)		
	University	20 (6.7)	24 (8.0)	40 (13.3)	20 (6.7)	2 (0.7)	1 (0.3)		
Type III	Illiterate	20 (6.7)	13 (4.3)	8 (2.7)	2 (0.7)	6 (2.0)	2 (0.7)	42.44	0.038**
	Primary	14 (4.7)	9 (3.0)	8 (2.7)	1 (0.3)	1 (0.3)	2 (0.7)		
	Middle school	19 (6.3)	9 (3.0)	9 (3.0)	8 (2.7)	5 (1.7)	1 (0.3)		
	High school	13 (4.3)	10 (3.3)	15 (5.0)	6 (2.0)	7 (2.3)	5 (1.7)		
	University	13 (4.3)	29 (9.7)	31 (10.3)	23 (7.7)	9 (3.0)	2 (0.7)		
Type IV	Illiterate	22 (7.3)	6 (2.0)	7 (2.3)	5 (1.7)	9 (3.0)	2 (0.7)	44.06	0.028**
	Primary	15 (5.0)	3 (1.0)	3 (1.0)	4 (1.3)	9 (3.0)	1 (0.3)		
	Middle school	17 (5.7)	3 (1.0)	14 (4.7)	7 (2.3)	5 (1.7)	5 (1.7)		
	High school	8 (2.7)	10 (3.3)	12 (4.0)	7 (2.3)	11 (3.7)	8 (2.7)		
	University	13 (4.3)	20 (6.7)	19 (6.3)	19 (6.3)	26 (8.7)	10 (3.3)		
Type V	Illiterate	26 (8.7)	7 (2.3)	6 (2.0)	3 (1.0)	4 (1.3)	5 (1.7)	52.19	0.005**
	Primary	15 (5.0)	6 (2.0)	7 (2.3)	2 (0.7)	4 (1.3)	1 (0.3)		
	Middle school	15 (5.0)	7 (2.3)	13 (4.3)	4 (1.3)	5 (1.7)	7 (2.3)		
	High school	11 (3.7)	5 (1.7)	8 (2.7)	8 (2.7)	14 (4.7)	10 (3.3)		
	University	15 (5.0)	11 (3.7)	17 (5.7)	22 (7.3)	29 (9.7)	13 (4.3)		
Male model									
Type II	Illiterate	29 (9.7)	8 (2.7)	3 (1.0)	3 (1.0)	5 (1.7)	3 (1.0)	43.77	0.031**
	Primary	18 (6.0)	8 (2.7)	4 (1.3)	3 (1.0)	1 (0.3)	1 (0.3)		
	Middle school	25 (8.3)	10 (3.3)	8 (2.7)	4 (1.3)	4 (1.3)	0 (0.0)		
	High school	19 (6.3)	15 (5.0)	8 (2.7)	9 (3.0)	0 (0.0)	5 (1.7)		
	University	30 (10.0)	31 (10.3)	26 (8.7)	16 (5.3)	3 (1.0)	1 (0.3)		
Type III	Illiterate	22 (7.3)	5 (1.7)	8 (2.7)	7 (2.3)	9 (3.0)	0 (0.0)	52.60	0.005**
	Primary	16 (5.3)	5 (1.7)	5 (1.7)	5 (1.7)	2 (0.7)	2 (0.7)		
	Middle school	22 (7.3)	10 (3.3)	10 (3.3)	6 (2.0)	3 (1.0)	0 (0.0)		
	High school	13 (4.3)	17 (5.7)	14 (4.7)	8 (2.7)	0 (0.0)	4 (1.3)		
	University	19 (6.3)	36 (12.0)	30 (10.0)	14 (4.7)	7 (2.3)	1 (0.3)		
Type IV	Illiterate	21 (7.0)	8 (2.7)	9 (3.0)	3 (1.0)	5 (1.7)	5 (1.7)	37.71	0.088*
	Primary	17 (5.7)	6 (2.0)	5 (1.7)	4 (1.3)	2 (0.7)	1 (0.3)		
	Middle school	19 (6.3)	10 (3.3)	8 (2.7)	4 (1.3)	4 (1.3)	6 (2.0)		
	High school	17 (5.7)	6 (2.0)	16 (5.3)	6 (2.0)	6 (2.0)	5 (1.7)		
	University	13 (4.3)	27 (9.0)	30 (10.0)	13 (4.3)	18 (6.0)	6 (2.0)		
Type V	Illiterate	18 (6.0)	7 (2.3)	12 (4.0)	4 (1.3)	4 (1.3)	6 (2.0)	25.78	0.466*
	Primary	13 (4.3)	7 (2.3)	6 (2.0)	2 (0.7)	6 (2.0)	1 (0.3)		
	Middle school	16 (5.3)	9 (3.0)	9 (3.0)	8 (2.7)	4 (1.3)	5 (1.7)		
	High school	11 (3.7)	11 (3.7)	14 (4.7)	4 (1.3)	11 (3.7)	5 (1.7)		
	University	16 (5.3)	18 (6.0)	24 (8.0)	20 (6.7)	17 (5.7)	12 (4.0)		

Participants with lower education levels more frequently selected lighter shades regardless of skin tone. In contrast, university-educated participants showed a tendency to select lighter shades for lighter skin tones and relatively darker shades for darker skin tones. However, effect sizes were small, with Cramer's V ranging from 0.19 to 0.21, indicating weak associations despite statistical significance.

Influence of professional status

No statistically significant association was observed between professional status and tooth shade preference for either the female or male model across Fitzpatrick skin types II through V (all p > 0.05) (Table 6). Government employees, private employees, and unemployed participants all showed a consistent preference for lighter tooth shades (BL1-BL3), regardless of the model's skin tone. These findings suggest that occupational category does not meaningfully influence esthetic tooth shade perception in this Moroccan sample. This result is consistent with the overall pattern observed across other demographic variables in this study, in which lighter shades were predominantly favored, irrespective of the observer's socioeconomic or professional background.

**Table 6:** Distribution of tooth shade selections by Fitzpatrick skin type, observer gender, and professional status, with chi-square test results. *P-value calculated using chi-square with Monte Carlo permutation test (B = 100,000 simulations) due to expected cell counts < 5 in one or more cells (affected groups: female model types II and III, min E = 2.2-2.6; male model types II, III, and IV, min E = 1.7-5.0). Data are presented as n (%). Statistical comparisons across professional status categories (governmental employee, private employee, unemployed) within each gender and Fitzpatrick skin type category were performed using Pearson's chi-square test (df = 10). No statistically significant association was found between professional status and preferred dental shade for any skin type or model (all p > 0.05). BL1, BL2, BL3, BL4, B1, and A1 refer to tooth shade tabs from the Ivoclar Vivadent A-D shade guide. min E: minimum expected cell count in the contingency tables

Model/skin type	Professional status	BL1, n (%)	BL2, n (%)	BL3, n (%)	BL4, n (%)	B1, n (%)	A1, n (%)	χ²	p-Value
Female model									
Type II	Governmental employee	19 (6.3)	23 (7.7)	32 (10.7)	15 (5.0)	4 (1.3)	2 (0.7)	21.20	0.150*
	Private employee	22 (7.3)	8 (2.7)	18 (6.0)	8 (2.7)	7 (2.3)	2 (0.7)		
	Unemployed	59 (19.7)	25 (8.3)	30 (10.0)	15 (5.0)	5 (1.7)	6 (2.0)		
Type III	Governmental employee	13 (4.3)	23 (7.7)	28 (9.3)	17 (5.7)	9 (3.0)	5 (1.7)	14.92	0.418*
	Private employee	18 (6.0)	16 (5.3)	13 (4.3)	9 (3.0)	6 (2.0)	3 (1.0)		
	Unemployed	48 (16.0)	31 (10.3)	30 (10.0)	14 (4.7)	13 (4.3)	4 (1.3)		
Type IV	Governmental employee	15 (5.0)	17 (5.7)	16 (5.3)	11 (3.7)	23 (7.7)	13 (4.3)	14.38	0.157
	Private employee	17 (5.7)	7 (2.3)	12 (4.0)	9 (3.0)	14 (4.7)	6 (2.0)		
	Unemployed	43 (14.3)	18 (6.0)	27 (9.0)	22 (7.3)	23 (7.7)	7 (2.3)		
Type V	Governmental employee	15 (5.0)	11 (3.7)	16 (5.3)	15 (5.0)	24 (8.0)	14 (4.7)	14.97	0.133
	Private employee	21 (7.0)	5 (1.7)	13 (4.3)	9 (3.0)	9 (3.0)	8 (2.7)		
	Unemployed	45 (15.0)	21 (7.0)	22 (7.3)	15 (5.0)	25 (8.3)	12 (4.0)		
Male model									
Type II	Governmental employee	35 (11.7)	19 (6.3)	21 (7.0)	13 (4.3)	3 (1.0)	4 (1.3)	10.91	0.678*
	Private employee	26 (8.7)	15 (5.0)	12 (4.0)	5 (1.7)	3 (1.0)	4 (1.3)		
	Unemployed	60 (20.0)	38 (12.7)	16 (5.3)	17 (5.7)	7 (2.3)	2 (0.7)		
Type III	Governmental employee	23 (7.7)	28 (9.3)	20 (6.7)	15 (5.0)	4 (1.3)	5 (1.7)	14.93	0.415*
	Private employee	23 (7.7)	10 (3.3)	16 (5.3)	9 (3.0)	4 (1.3)	3 (1.0)		
	Unemployed	46 (15.3)	35 (11.7)	31 (10.3)	16 (5.3)	12 (4.0)	0 (0.0)		
Type IV	Governmental employee	18 (6.0)	18 (6.0)	21 (7.0)	15 (5.0)	16 (5.3)	7 (2.3)	20.06	0.184*
	Private employee	17 (5.7)	14 (4.7)	14 (4.7)	6 (2.0)	5 (1.7)	9 (3.0)		
	Unemployed	52 (17.3)	25 (8.3)	33 (11.0)	9 (3.0)	14 (4.7)	7 (2.3)		
Type V	Governmental employee	18 (6.0)	10 (3.3)	23 (7.7)	14 (4.7)	17 (5.7)	13 (4.3)	14.37	0.157
	Private employee	14 (4.7)	11 (3.7)	14 (4.7)	9 (3.0)	11 (3.7)	6 (2.0)		
	Unemployed	42 (14.0)	31 (10.3)	28 (9.3)	15 (5.0)	14 (4.7)	10 (3.3)		

Grouped shade analysis

When tooth shades were grouped into lighter shades (BL1-BL4) and conventional shades (A1-B1), lighter shades were significantly more frequently selected across Fitzpatrick skin types II through V for both models (all χ² tests, p < 0.001). The proportion of lighter shade selections ranged from approximately 69% to 92%. This grouped analysis statistically confirmed a strong overall preference for lighter tooth shades irrespective of model skin tone or observer characteristics. Overall, lighter shades were consistently preferred across all analyses, whereas demographic variables showed limited influence on shade selection.

## Discussion

Shade matching is a complex process that involves both subjective and objective factors. The success of esthetic treatments largely depends on selecting the right tooth shade, a task that remains challenging for practitioners. However, patients' desires often do not align with reality, as they are influenced by societal misconceptions and external factors. These beliefs shape public perception, complicating the process of choosing the most suitable shade for each individual.

In our study concerning the gender parameter, our findings align with previous studies indicating that gender does not significantly influence the choice of tooth shade [3,5,11,12]. Both male and female participants consistently selected lighter shades (BL1, BL2, and BL3), suggesting that the prevailing trend toward whiter teeth is not gender specific. This preference could be attributed to external influences, such as the impact of social media, celebrity endorsements, and fashion trends, all of which have contributed to the increasing demand for lighter tooth shades.

Regarding age, our sample demonstrated a clear preference for lighter tooth shades, regardless of participants' ages. This trend reflects a broader societal shift towards brighter dental esthetics, with whiter teeth being perceived as more desirable [5,8]. However, the study by Vallittu et al. found that the perception of extremely white teeth decreased with age [13]. This discrepancy can likely be attributed to the fact that older individuals may be less influenced by short-term trends and tend to favor more natural tooth colors that are in harmony with their personal identity and age.

A non-significant correlation was found between skin color and the choice of tooth shade in our findings, consistently showing that the lightest shades (BL1, BL2, and BL3) were preferred, as reported by several previous studies [11,12,14]. However, this contrasts with the findings of Labban et al. in Saudi Arabia, who observed a general trend in which individuals with lighter skin tones preferred lighter tooth shades, whereas those with darker skin tones favored darker shades [8]. Similar trends have been reported in other studies conducted in various countries [15-18].

However, contradicting findings have emerged from other research, which suggest that individuals with darker skin tones may prefer lighter tooth shades, while those with lighter skin tones tend to choose darker shades [3]. These conflicting results highlight that the choice of tooth shade is likely influenced by a range of factors beyond skin color, including cultural, psychological, and individual preferences.

Additionally, we observed a significant association between education level and tooth shade preference for most subgroups in our sample, which is consistent with findings from Vallittu et al. [13]. This association was statistically significant for the female model across all four skin types and for the male model with lighter skin tones (Fitzpatrick types II and III). However, no significant association was found for the male model with darker skin tones (types IV and V), possibly reflecting greater variability in esthetic preferences when evaluating darker complexions, or reduced statistical power due to the sparse distribution of responses across some education-level and shade combinations. Among participants who showed a significant preference pattern, those with higher levels of education were more likely to associate lighter dental shades with lighter skin tones and darker shades with darker skin tones. In contrast, those with lower levels of education tended to consistently select lighter, often bleached tooth shades, regardless of skin tone. This trend could be linked to the perception that whiter teeth symbolize higher social status, a notion that is frequently reinforced through media portrayals [8].

Although this study provides insightful data on the association between demographic factors and tooth shade preferences, several limitations must be acknowledged. The study population was composed exclusively of Moroccan participants, which, while valuable for understanding local trends, may limit the generalizability of the findings to broader or more diverse populations. Moreover, the reliance on digitally displayed images for tooth shade selection, although methodologically convenient, may not fully replicate real-life perception due to variability in screen calibration, lighting conditions, and other environmental factors. The use of only two models may also limit generalizability and introduce bias toward facial morphology. Overall, more robust conclusions would require a multifactorial and ecologically valid study design that better reflects real-life dental esthetic perception. Such an approach should integrate heterogeneous populations, standardized assessment tools for skin tone and tooth shade, and consider the influence of cultural, psychological, and socioeconomic factors on esthetic preferences.

## Conclusions

This study revealed that the Moroccan population in our sample did not make a clear association between skin tone and dental shade selection. Instead, participants consistently favored lighter, brighter teeth regardless of skin color, suggesting that a culturally ingrained ideal of dental esthetics, rooted in the perception of perfect teeth as being white and pearly, persists. This tendency reflects a preference pattern where lighter teeth are often linked to positive social judgments, including competence, attractiveness, and intellectual ability. However, such preferences are not universal; they vary according to ethnic background, lifestyle, and educational level. Therefore, while our findings highlight a clear esthetic preference among this Moroccan population, they cannot be generalized to other ethnic groups without further investigation. Future studies should include diverse populations and explore the multidimensional factors, such as cultural, educational, and psychological influences that shape dental shade preferences, in order to develop more inclusive and patient-centered esthetic guidelines.

## Appendices

Questionnaire

This study aimed to evaluate the influence of gender and skin color on tooth shade preferences. This questionnaire is strictly anonymous. Thank you for taking the time to complete it.

Appendix 1

Please answer each question by checking the appropriate box or indicating the number that best reflects your opinion, taking into account your gender, age, education level, occupation, and income, as well as your level of satisfaction with your smile and the importance you place on it.

**Table 7:** Sociodemographic characteristics and smile satisfaction questionnaire. MAD: Moroccan Dirham

Item	Question	Response options
1	Gender	☐ Female ☐ Male
2	Age	_____ years
3	Education level	_____
4	Occupation	_____
5	Income (MAD)	☐ 0-2500 ☐ 2501-4166 ☐ 4167-5000 ☐ 5001-6666 ☐ 6667-15000 ☐ ≥ 15001
6	Are you satisfied with your smile?	0 1 2 3 4 5 6 7 8 9 10 (0 = not satisfied at all; 10 = completely satisfied)
7	Is your smile important to you?	0 1 2 3 4 5 6 7 8 9 10 (0 = not important at all; 10 = extremely important)

Appendix 2

Please check the box corresponding to the photo you find most esthetically pleasing, taking into account skin color and gender (for each skin color, please choose only one photo).

**Table 8:** Distribution of tooth shade preferences for the female model across different Fitzpatrick skin phototypes.

Skin type	BL1	BL2	BL3	BL4	A1	B1
Type II	☐	☐	☐	☐	☐	☐
Type III	☐	☐	☐	☐	☐	☐
Type IV	☐	☐	☐	☐	☐	☐
Type V	☐	☐	☐	☐	☐	☐

**Table 9:** Distribution of tooth shade preferences for the male model across different Fitzpatrick skin phototypes.

Skin type	BL1	BL2	BL3	BL4	A1	B1
Type II	☐	☐	☐	☐	☐	☐
Type III	☐	☐	☐	☐	☐	☐
Type IV	☐	☐	☐	☐	☐	☐
Type V	☐	☐	☐	☐	☐	☐
